# Association between Loneliness and Diseases Self-Management in Older Adults: Systematic Review

**DOI:** 10.1093/geroni/igab046.2380

**Published:** 2021-12-17

**Authors:** Emma Cho, Alexandra Garcia, Ya-Ching Huang, Hsuan-Ju Kuo

**Affiliations:** 1 University of Texas at Austin School of Nursing, Austin, Texas, United States; 2 The University of Texas at Austin, Austin, Texas, United States; 3 Texas State University, Round Rock, Texas, United States

## Abstract

Purpose: Older adults with chronic diseases are more at risk for loneliness, and loneliness has a negative impact on health behaviors, which are key to managing chronic diseases. However, little is known about the association between loneliness and self-management behaviors in older adults with chronic diseases. As societies worldwide experience the growth of aging populations who are at higher risk of having chronic diseases as they age, clinicians and researchers should assess and address loneliness of older adults with chronic diseases. Methods: This systematic review synthesizes research found in PubMed, MEDLINE, PsychINFO, CINAHL, and SocINDEX. Findings: fourteen studies were conducted in four countries and represented n= 128,610. Loneliness was measured by three different instruments. Reports of loneliness were frequent and ranged from 7.7% (in a report of severe loneliness) to 43.2% (moderate loneliness) of older adults. Older adults who experienced loneliness were less likely to be physically active, eat a healthy diet, or cope in positive ways and more likely to be female and seek healthcare. Conclusions: This systematic review found that loneliness was moderately prevalent, and that loneliness was associated with negative disease self-management behaviors in older adults with chronic diseases. Gaps in the research include a need for studies guided by theoretical pathways, using a consistent, theoretically-based measure of loneliness, and conducted on among people with specific chronic diseases.

